# SIRT3 mediates the effects of PCSK9 inhibitors on inflammation, autophagy, and oxidative stress in endothelial cells

**DOI:** 10.7150/thno.80289

**Published:** 2023-01-01

**Authors:** Nunzia D'Onofrio, Francesco Prattichizzo, Raffaele Marfella, Celestino Sardu, Elisa Martino, Lucia Scisciola, Lorenza Marfella, Rosalba La Grotta, Chiara Frigé, Giuseppe Paolisso, Antonio Ceriello, Maria Luisa Balestrieri

**Affiliations:** 1 Department of Precision Medicine, the University of Campania “Luigi Vanvitelli” Italy.; 2 IRCCS MultiMedica, Via Fantoli 16/15, 20138, Milan, Italy.; 3 Università degli Studi della Campania “Luigi Vanvitelli”, Piazza Luigi Miraglia 2, 80138, Naples, Italy.; 4 Mediterranea Cardiocentro, 80122, Naples, Italy.

**Keywords:** PCSK9, SIRT3, IL-6, endothelial cells, cardiovascular diseases, ROS, oxidative stress, inflammation, autophagy, LDL-cholesterol, inflammasome

## Abstract

**Background**: Proprotein convertase subtilisin-kexin type 9 (PCSK9) inhibitors (i) are a class of lipid-lowering drugs suggested to hold a plethora of beneficial effects independent of their LDL cholesterol-lowering properties. However, the mechanism underlying such observations is debated.

**Methods**: Human aortic endothelial cells (TeloHAEC) were pre-treated with 100 µg/mL of the PCSK9i evolocumab and then exposed to 20 ng/mL of IL-6, a major driver of cardiovascular diseases (CVD), in both naïve state and after siRNA-mediated suppression of the NAD-dependent deacetylase sirtuin-3 (SIRT3). Inflammation, autophagy, and oxidative stress were assessed through Western Blots, ELISAs, and/or immunofluorescence coupled by flow cytometry. To explore the human relevance of the findings, we also evaluated the expression of IL-6, SIRT3, IL-1β, the ratio LC3B II/I, and PCSK9 within the plaques of patients undergoing carotid endarterectomy (n=277), testing possible correlations between these proteins.

**Results**: PCSK9i improved a range of phenotypes including the activation of inflammatory pathways, oxidative stress, and autophagy. Indeed, treatment with PCSK9i was able to counteract the IL-6 induced increase in inflammasome activation, the accrual of autophagic cells, and mitochondrial ROS accumulation. Of note, silencing of SIRT3 reverted the beneficial effects observed with PCSK9i treatment on all these phenomena. In atheroma specimens, the expression of PCSK9 was inversely related to that of SIRT3 while positively correlating with IL-6, IL-1β, and the ratio LC3B II/I.

**Conclusions**: Overall, these data suggest that PCSK9i bear intrinsic anti-inflammatory, anti-autophagic, and antioxidant properties in endothelial cells, and that these pleiotropic effects might be mediated, at least in part, by SIRT3. These results provide an additional mechanism supporting the emerging knowledge relative to the benefit of PCSK9i on CVD beyond LDL-lowering and uncover SIRT3 as a putative mediator of such pleiotropy.

## Introduction

Proprotein convertase subtilisin-kexin type 9 (PCSK9) inhibitors (i) are a class of lipid-lowering drugs promoting a marked decrease in low-density lipoprotein (LDL)-cholesterol and demonstrated to reduce the incidence of cardiovascular diseases (CVD) in patients not-at-target with other therapies or intolerant to statins 1. Post-hoc analyses of trials suggested a potential benefit on CVD independent of LDL-cholesterol reduction 2, while preclinical data evidenced a plethora of possible beneficial effects of PCSK9i, which have been reported to ameliorate inflammation 3,4, autophagy 5,6, and oxidative stress 6, with these phenomena being independent of LDL-cholesterol levels. However, the mechanisms underlying such observations are unclear.

PCSK9 protein is mainly expressed in the liver where it operates the removal of LDL receptors 7. However, a number of reports demonstrated its expression in multiple tissues, including in endothelial cells (EC) 8. PCSK9 expression is closely intertwined with low-grade inflammation. Indeed, different pro-inflammatory stimuli induce an increase in PCSK9 expression 9, 10, which in turn fosters the activation of pro-inflammatory pathways 11,12. Plasma levels of PCSK9 are positively related to a range of pro-inflammatory mediators 12-14, including interleukin (IL)-6 15. IL-6 is a cytokine increasingly recognized as a major driver of cardiovascular diseases (CVD) 16. Among other mechanisms, IL-6 have been suggested to promote deleterious effects on the vasculature through the induction of pro-inflammatory pathways, oxidative stress, and autophagy 17-19. However, whether PCSK9i are able to counteract the detrimental mechanisms instigated by IL-6 in EC is unknown.

Sirtuins (SIRT) are a family of nicotinamide adenine dinucleotide-(NAD+) dependent histone deacetylases involved in the regulation of a range of homeostatic processes 20. SIRT3 is a key regulator of mitochondrial lysine acetylation 21, albeit its expression has been reported also in the cytoplasm or even in the nucleus 22. Among other functions, SIRT3 has been suggested as a regulator of metabolic and antioxidant responses 23,24. Considering also its emerging role in the control of inflammatory pathways 25 and autophagy 26, SIRT3 has been proposed as a possible target to prevent or ameliorate CVD in a consistent number of preclinical studies 27,28. However, no report explored the role of SIRT3 in the context of the cardioprotective effects of PCSK9i.

In this study, we first evaluated a possible ability of PCSK9i to counteract the pro-inflammatory, pro-oxidant, and pro-autophagic effects of IL-6 in EC. Then, considering the knowledge relative to the ability of SIRT3 to blunt these phenomena 23-26, we explored a potential role for this protein in this context. Finally, we assessed the correlation between IL-6 and the expression of SIRT3, IL-1β, the ratio LC3B II/I, and PCSK9 within the plaques of patients undergoing carotid endarterectomy.

## Methods

### Cell culture and treatments

The human aortic endothelial cells (EC) (TeloHAEC cell line, CRL-4052, Lot#70027940) were grown as a monolayer in Vascular Cell Basal Medium (PCS-100-030) supplemented with Endothelial Cell Growth kit-VEGF (PCS-100-041) in incubator at 37 °C under a humidified atmosphere with 95% air and 5% CO_2_. EC, medium, and supplementation culture kit were all purchased from ATCC (American Type Culture Collection, Manassas, VA, USA). To induce inflammatory stress, EC were treated for a maximum time of 24h with increasing concentrations (5-20 ng/mL) of IL-6. The PCSK9i, evolocumab, under the commercial name of Repatha, was a human monoclonal antibody inhibiting Proprotein Convertase Subtilisin/Kexin type 9 (PCSK9) provided by Amgen Europe B.V. A PCSK9i stock solution was obtained by diluting the pharmaceutical solution (140 mg/mL) in Hanks' balanced salt solution (HBSS)-10 mM Hepes and treatments on EC performed with increasing concentrations (10-100 µg/mL) up to 72h. For the combined treatment (PCSK9i+IL-6), EC were pre-treated for 8h with 100 µg/mL PCSK9i before being exposed to 20 ng/mL IL-6 for 24h. Control cells (Ctr) were maintained in a complete culture medium with the corresponding highest volume of HBSS-10 mM Hepes.

### Cell viability

EC were seeded in 96-well plates and, after treatments, viability detected using Cell Counting Kit-8 (CCK-8, Donjindo Molecular Technologies, Inc., Rockville, MD, USA), following the manufacturer's instructions. Briefly, CCK-8 solution (10 μL) was added to each well and then plated and incubated at 37 °C for 4h. Absorbance was measured at 450 nm using a microplate reader (model 680, Bio-Rad, Hercules, CA, USA) and viability expressed as percentage of control. All experiments were performed with n = 6 replicates.

### Mitochondrial ROS measurement

MitoSOX Red Mitochondrial Superoxide Indicator (M36008, Thermo Scientific, Rockford, IL, USA) was used to selectively assess the mitochondrial ROS content, following the manufacturer's protocols. After treatment, EC were stained with 5 μM Mitosox for 20 min at 37 °C in complete medium. Cells were imaged on a fluorescence microscope EVOS FL Cell Imaging System (Thermo Scientific Rockford, IL, USA,) and then detached, washed in phosphate-buffered saline (PBS) and the median fluorescence intensity (MFI) quantified using a BD Accuri C6 cytometer (BD Biosciences, San José, CA, USA). Data were analyzed by FlowJo V10 software (Williamson Way, Ashland, OR, USA). Treatment for 30 min with 50 μM menadione (M57405, Sigma Aldrich, St. Louis, MO, USA,) was used as positive control (**Supplementary Figure 1A, B**).

### Mitochondrial membrane potential

Mitochondrial membrane potential was determined by using JC-1 staining (MT09, Dojindo Molecular Technologies, Tokyo, Japan), according to the manufacturer's protocols. After treatments, EC were stained for 1h with 5 µM JC-1 probe at 37 °C, imaged on a fluorescence microscope EVOS FL Cell Imaging System (Thermo Scientific, Rockford, IL, USA) and then fluorescence registered with BD Accuri C6 (BD Biosciences, San José, CA, USA) cytometer. Results were analyzed by FlowJo V10 software (Williamson Way, Ashland, OR, USA).

### Autophagy detection

Autophagy was detected by staining for 30 min treated EC with 1 µM Autophagy Assay Kit (ab139484, Abcam, Cambridge, UK) at 37 °C in the dark, as manufacturer's indication. Cells were then washed with PBS and imaged on a fluorescence microscope EVOS FL Cell Imaging System (Thermo Scientific, Rockford, IL, USA), before FACS analysis. Treatment with 1 µM rapamycin overnight was used as positive control. Flow cytometry evaluation was performed using a BD Accuri C6 instrument (BD Biosciences, San José, CA, USA) and data analyzed by FlowJo V10 software (Williamson Way, Ashland, OR, USA). For each sample at least 20,000 events were recorded. EC treated with rapamycin were used as positive control (**Supplementary Figure 1C, D**).

### Cell lysis and immunoblotting

EC lysates were obtained in lysis buffer containing 1% NP-40, 0.5% sodium deoxycholate, 0.1% SDS, 10 μg/mL aprotinin, leupeptin and 1 mM phenylmethylsulfonylfluoride in PBS. Proteins (40-80 μg) were separated by sodium dodecyl sulfate-polyacrylamide gel electrophoresis (SDS-PAGE) and then transferred to nitrocellulose membranes. Blocking of non-specific binding sites was obtained by incubating blots for 1h in 1× TBS 1% casein blocker (1610782, Bio-Rad, Hercules, CA, USA) at room temperature. Then, membranes were incubated overnight at 4 °C with specific primary antibodies: anti-Proprotein Convertase Subtilisin/Kexin type 9 (PCSK9, 1:1000, ab28770, Abcam, Cambridge, UK); anti-tumour necrosis factor alpha (TNF-α, 1:1000, ab6671, Abcam, Cambridge, UK), anti-nuclear factor kappa B (NF-κB, 1:2000, ab16502, Abcam, Cambridge, UK); anti-SIRT3 (1:2000, PA5-86035, Invitrogen, Waltham, MA, USA); anti-NLR Family Pyrin Domain Containing 3 (NLRP3,1:1000, ab270449, Abcam, Cambridge, UK), anti-caspase-1 (1:500, sc-56036, Santa Cruz Biotechnology, Dallas, TX, USA); anti-IL-1β (1:1000, ab216995, Abcam, Cambridge, UK); anti-autophagy related 5 (ATG5, 1:1000, 9980, Cell Signaling Technology, Danvers, MA, USA); anti-beclin-1 (1:1000, 4122, Cell Signaling Technology, Danvers, MA, USA); anti-sequestosome-1 (SQSTM1/p62, 1:2000, 5114, Cell Signaling Technology, Danvers, MA, USA); anti-microtubule-associated protein 1 light chain 3B (LC3B, 1:2000, ab192890, Abcam, Cambridge, UK); anti-α-tubulin (1:5000, E-AB-20036, Elabscience Biotechnology Inc., Houston, TX, USA); and anti-glyceraldehyde-3-phosphate dehydrogenase (GAPDH, 1:2000, ab9485, Abcam, Cambridge, UK). After 1h incubation with peroxidase-conjugated secondary antibodies, immunocomplexes were revealed on dried membranes by using the Excellent Chemiluminescent Substrate kit (E-IR-R301, Elabscience Biotechnology Inc., Houston, TX, USA) and acquired by ChemiDoc Imaging System with Image Lab 6.0.1 software (Bio-Rad Laboratories, Milan, Italy). After background subtraction, the densities of immunoreactive bands, were quantified by ImageJ software 1.52n version (Wayne Rasband, National Institutes of Health, USA) and reported as arbitrary units (AU).

### ELISA assays

Levels of NLRP3, caspase-1 and of cytokines (IL-1β, TNF-α and IFN-γ) were all detected by ELISA assays (human NLRP3 ELISA kit, ab274401, Abcam, Cambridge, UK; human caspase-1 ELISA kit, ab219633, Abcam, Cambridge, UK; human IL-1β quantikine ELISA, DLB50, R&D Systems, Inc., Minneapolis, MN, USA; human TNF alpha ELISA kit, ab181421, Abcam, Cambridge, UK; human IFN-gamma ELISA, RAF021R, BioVendor Laboratorni medicina a.s., Brno, Czech Republic), according to the manufacturer's protocols. EC lysates or supernatants (50-200 µL) were incubated for 1-2h in precoated plates with specific cocktail or anti-cytokine antibodies. After 3 time washing, development or substrate solutions were added and plate incubated for 10-20 min in the dark, before the addition of stop solution to each well. The absorbance was detected with a microplate reader (model 680, Bio-Rad, Hercules, CA, USA) at 450 nm and NLRP3, caspase-1, and cytokine levels determined by plotting the absorbance values against each standard curve concentrations.

### SiRNA mediated gene silencing

For transient SIRT3 silencing, EC were transfected with 30 nM control non-targeting siRNA (NT^-/-^) or with SIRT3 siRNA Oligos set for specific human Sirtuin 3 (SIRT3^-/-^, 438080910101, Applied Biological Materials, Inc., Richmond, BC, Canada), in serum- and antibiotic-free medium, using Lipofectamin (vehicle) as transfection reagent. EC were incubated for 8h, followed by additional 12h of incubation after the addition of FBS, before treatments.

### Cohort description and quantitation of proteins in atheroma specimens

Patients with carotid stenosis (according to North American Symptomatic Carotid Endarterectomy Trial classification) enlisted to undergo carotid endarterectomy for extracranial high-grade (>70%) internal carotid artery stenosis were enrolled. Study design was as previously described 49, while clinical characteristics of the patients are reported in **Supplementary Table 1**. From the overall cohort, 277 random patients were selected for this study. Based on previous literature 50, we considered this sample size as adequate to explore possible correlations. Patients on treatment with PCSK9i were excluded from this analysis to avoid the confounding effect of the drug, while no other exclusion criteria were applied. The study was approved by the local Ethics Review Committee and informed written consent was obtained for each patient. Specimens obtained from atherectomy were frozen in liquid nitrogen for subsequent analyses. Protein extract samples from plaques were prepared by adding 300 µL of 2D lysis buffer (7 mol/L urea, 2 mol/L thiourea, 4% CHAPS (3-((3-cholamidopropyl) dimethylammonio)-1-propane sulfonate) buffer, 30 mmol/L Tris-HCl, pH 8.8) to tissues (50 mg) cut into small pieces. Tissue was homogenized with a Precellys 24 system (Bertin Technologies) and then centrifuged at 800xg for 10 min at 4°C to collect the supernatant. 40-70 μg of sample proteins were separated by sodium dodecyl sulfate-polyacrylamide gel electrophoresis (SDS-PAGE) and then transferred to nitrocellulose membranes. Then, membranes were incubated overnight at 4 °C with specific primary antibodies: anti-Proprotein Convertase Subtilisin/Kexin type 9 (1:1000, ab28770, Abcam, Cambridge, UK); anti-IL-6 (1:1000, #12153, Cell Signalling, Danvers, Massachusetts, USA); anti-SIRT3 (1:2000, PA5-86035, Invitrogen, Waltham, MA, USA); anti-IL-1β (1:1000, ab216995, Abcam); anti-microtubule-associated protein 1 light chain 3B (LC3B, 1:2000, ab192890); anti-α-tubulin (1:5000, E-AB-20036, Elabscience Biotechnology Inc., Houston, TX, USA); anti-actin (1:3000, ab179467, Abcam), and anti-glyceraldehyde-3-phosphate dehydrogenase (GAPDH, 1:2000, ab9485, Abcam). After 1h incubation with peroxidase-conjugated secondary antibodies, immunocomplexes were revealed on dried membranes by using the Excellent Chemiluminescent Substrate kit (E-IR-R301, Elabscience Biotechnology Inc.) and acquired by ChemiDoc Imaging System with Image Lab 6.0.1 software (Bio-Rad Laboratories, Milan, Italy). After background subtraction, the densities of immunoreactive bands were quantified by ImageJ software 1.52n version (Wayne Rasband, National Institutes of Health, USA) and reported as arbitrary units (AU).

### Statistical analysis

Data are presented as mean±SD. Continuous variables were compared with one-way ANOVA for normally distributed data and Kruskal-Wallis for non-normally distributed data. When differences among the groups were found, Bonferroni correction to make pairwise comparisons was used. Linear regression analyses were performed to estimate the relationship among IL-6, SIRT3, IL-1β, the ratio LC3B II/I, and PCSK9. P < 0.05 was considered statistically significant. All calculations were performed using SPSS 12. For *in vitro* studies, analyses and graphs were performed using GraphPad Prism version 9.1.2.

## Results

### PCSK9i attenuate IL-6 induced inflammation

**Figure 1:**
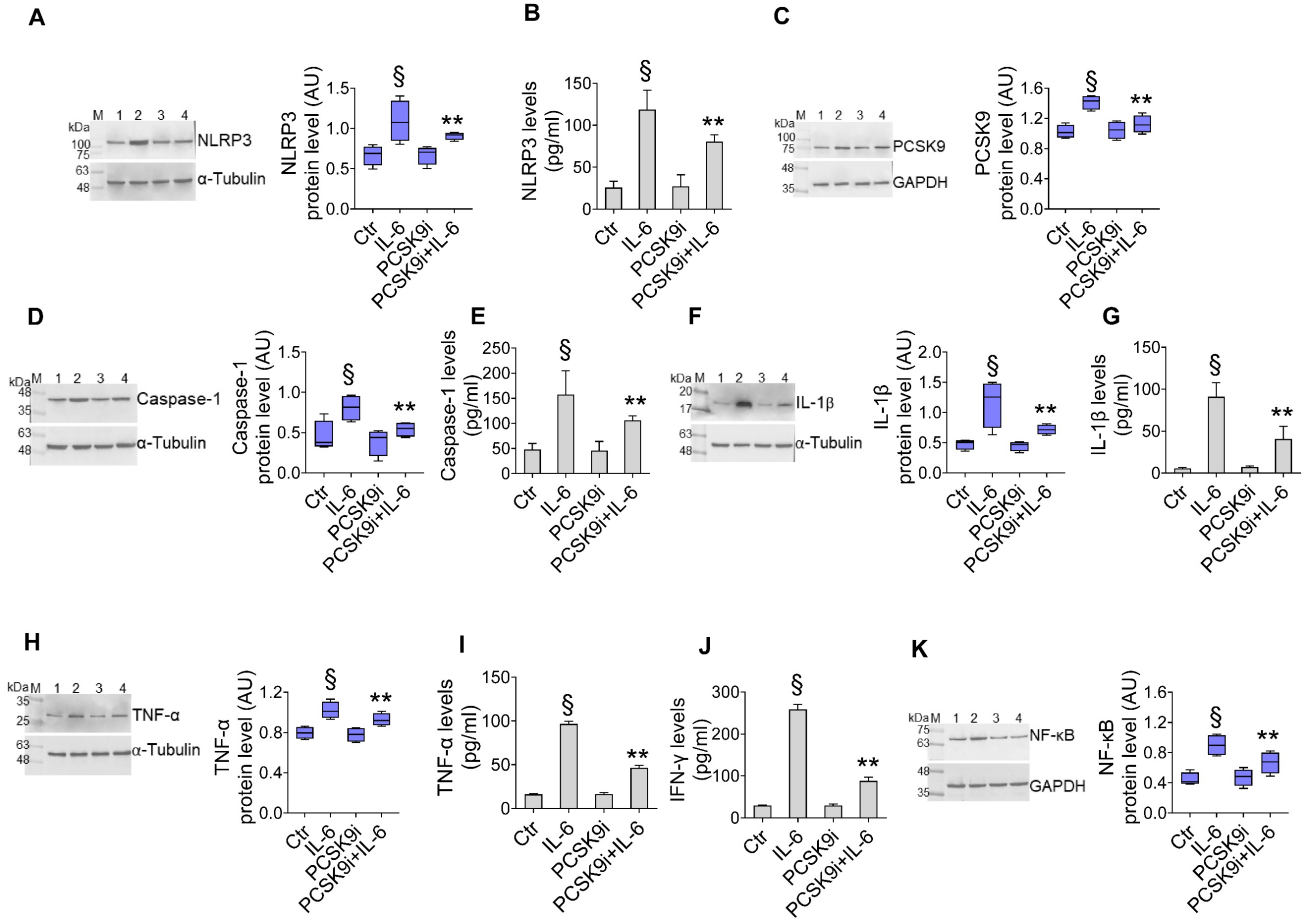
**PCSK9i counteract IL-6-induced inflammation**. Evaluation of **(A)** NLRP3 protein expression and **(B)** content,** (C)** PCSK9 protein expression, caspase-1 **(D)** expression and **(E)** content, IL-1β** (F)** expression and **(G)** content, TNF-α **(H)** expression and **(I)** content,** (J)** IFN-γ levels and **(K)** NF-κB expression levels in EC treated for 24h with 20 ng/mL IL-6 (IL-6), with 100 µg/mL PCSK9i (PCSK9i) or pre-treated for 8h with 100 µg/mL PCSK9i before being exposed to 20 ng/mL IL-6 for 24h (PCSK9i+IL-6). Control cells (Ctr) were maintained in complete culture medium with the corresponding highest volume of HBSS-10 mM Hepes. Immunoblotting analyses are represented as floating bars with line representing the mean of n = 4 independent experiments ± SD, where the densitometric intensity was calculated with ImageJ software and expressed as arbitrary units (AU). Lane 1 = Ctr; lane 2 = IL-6; lane 3 = PCSK9i; lane 4 = PCSK9i+IL-6; M = weight markers (G266, Applied Biological Materials Inc.). §p < 0.001 *vs.* Ctr; **p < 0.01 *vs.* IL-6.

We first assessed the effect of different dosages of IL-6 with and PCSK9i on human aortic endothelial cells (TeloHAEC) proliferation, a commonly used model of EC. Dose-response experiments indicated that treatment with IL-6 resulted in cytotoxic effects after 24h treatment with 20 ng/mL (**Supplementary Figure 2A**). On the contrary, incubation with evolocumab, a PCSK9i employed in clinical practise and with proven cardiovascular benefit 29, did not affect the viability of EC from 10 to 100 µg/mL up to 72h (**Supplementary Figure 2B**), while pre-treatment with 100 µg/mL PCSK9i for 8h showed protective effects on EC exposed to inflammatory IL-6 (**Supplementary Figure 2C**), therefore these concentrations were used for subsequent experiments. To explore whether PCSK9i are able to counteract the activation of inflammatory pathways instigated by IL-6, we pre-treated EC for 8h with 100 µg/mL PCSK9i before stimulating cells with 20 ng/mL IL-6 for an additional 24h. Treatment with IL-6 significantly increased the expression of NLRP3 (**Figure 1A**), PCSK9 (**Figure 1C**), caspase-1 (**Figure 1D**), IL-1β (**Figure 1F**), TNFα (**Figure 1H**), and NF-kB (**Figure 1K**), while treatment with PCSK9i hampered the induction of these pro-inflammatory mediators, as assessed by Western Blot. These results were corroborated by ELISA which showed the same trend for NLRP3 (**Figure 1B**), caspase-1 (**Figure 1E**), IL-1β (**Figure 1G**), TNFα (**Figure 1I**), but also for IFN-γ levels (**Figure 1J**) secreted in the culture media, overall supporting an anti-inflammatory activity for PCSK9i in EC stimulated with IL-6.

### PCSK9i counteract IL-6 induced autophagy

**Figure 2:**
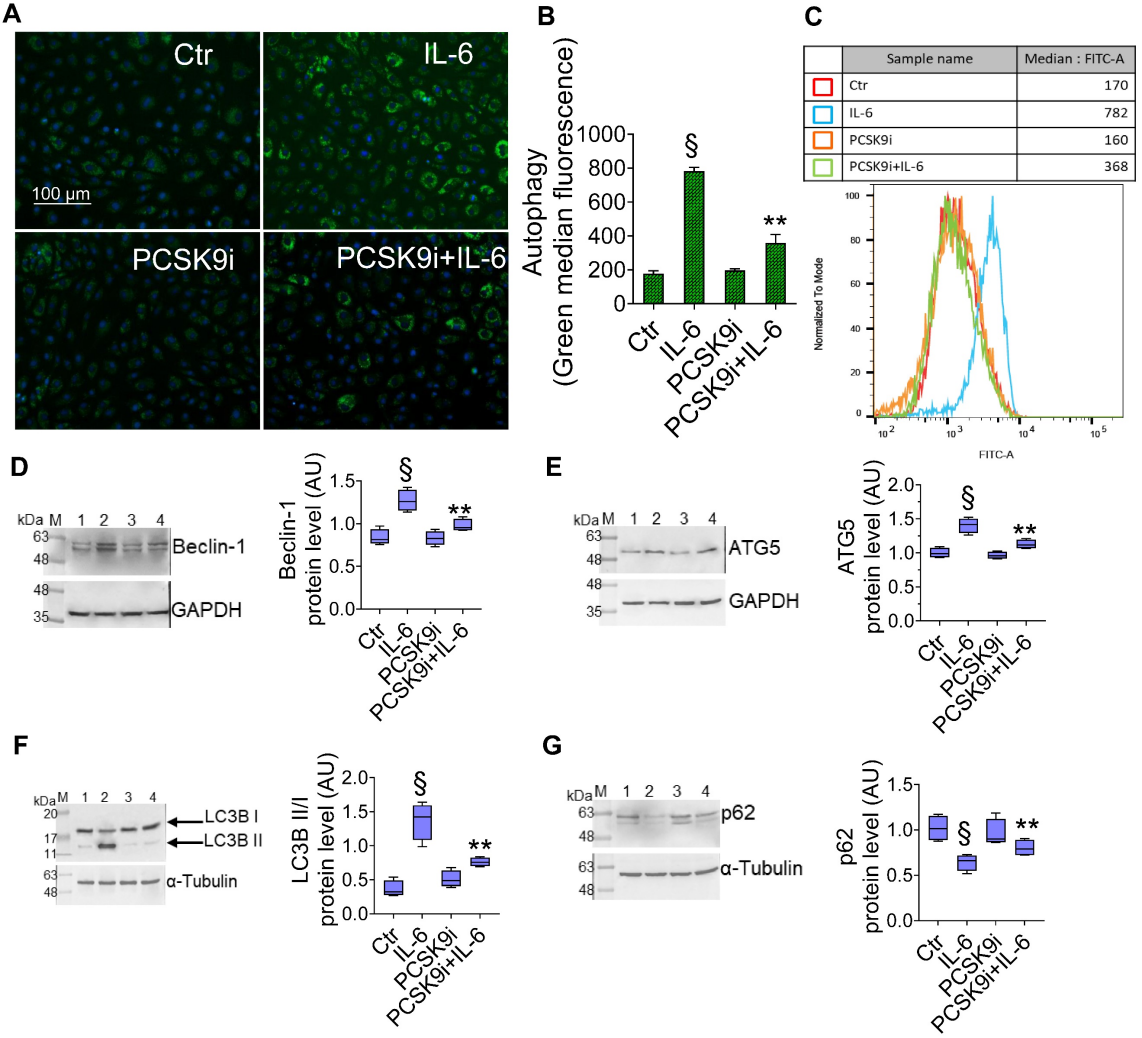
**PCSK9i decrease IL-6-related autophagy.** Representative images by fluorescence microscopy and flow cytometry analysis of **(A-C)** green detection reagent, quantified as median fluorescence intensities, and representative cropped blots with relative immunoblotting analysis of **(D)** beclin-1, **(E)** ATG5, **(F)** LC3B II/I, and **(G)** p62 protein levels in EC treated for 24h with 20 ng/mL IL-6 (IL-6), with 100 µg/mL PCSK9i (PCSK9i) or pre-treated for 8h with 100 µg/mL PCSK9i before being exposed to 20 ng/mL IL-6 for 24h (PCSK9i+IL-6). Control cells (Ctr) were maintained in complete culture medium with the corresponding highest volume of HBSS-10 mM Hepes. Scale bars = 100 µm. Lane 1 = Ctr; lane 2 = IL-6; lane 3 = PCSK9i; lane 4 = PCSK9i+IL-6; M = weight markers (G266, Applied Biological Materials Inc.). Immunoblotting analyses are represented as floating bars with line representing the mean of n = 4 independent experiments ± SD, where the densitometric intensity was calculated with ImageJ software and expressed as arbitrary units (AU). §p < 0.001 *vs.* Ctr; **p < 0.01 *vs.* IL-6.

To test whether PCSK9i are able to limit the induction of autophagy induced by IL-6, we used the same experimental setting to assess the autophagic flux in live EC through a dye that selectively labels autophagic vacuoles. IL-6 induced an accrual of autophagic cells, which was prevented by pre-treatment with PCSK9i (**Figure 2A-C**). Consistently, the expression of the pro-autophagic proteins Beclin-1 (**Figure 2D**), ATG5 (**Figure 2E**), and of the ratio LC3B II/I (**Figure 2F**) was increased by IL-6 and attenuated by PCSK9i, while the anti-autophagic p62 showed the opposite trend (**Figure 2G**).

### PCSK9i ameliorate ROS accrual and SIRT3 downregulation

**Figure 3:**
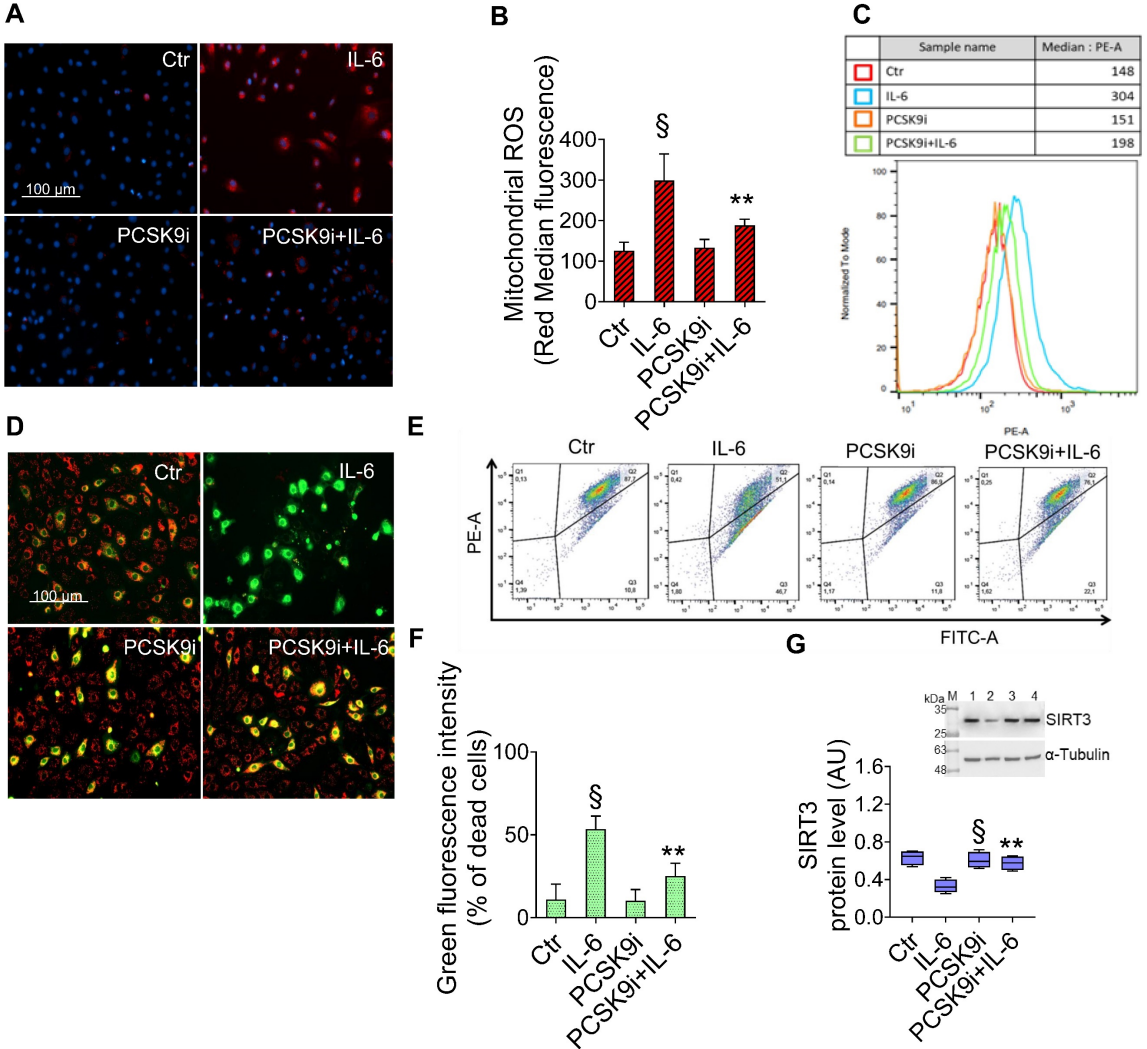
**PCSK9i inhibit IL-6-induced mitochondrial damage**. Representative images by fluorescence microscopy and FACS analysis of **(A-C)** mitochondrial ROS and **(D-F)** mitochondrial membrane potential detection, expressed as fluorescence intensity ± SD of n = 3 experiments, and **(G)** representative cropped blots with relative immunoblotting analysis of SIRT3 protein levels in EC treated for 24h with 20 ng/mL IL-6 (IL-6), with 100 µg/mL PCSK9i (PCSK9i) or pre-treated for 8h with 100 µg/mL PCSK9i before being exposed to 20 ng/mL IL-6 for 24h (PCSK9i+IL-6). Control cells (Ctr) were maintained in complete culture medium with the corresponding highest volume of HBSS-10 mM Hepes. Scale bars = 100 µm. Lane 1 = Ctr; lane 2 = IL-6; lane 3 = PCSK9i; lane 4 = PCSK9i+IL-6; M = weight markers (G266, Applied Biological Materials Inc.). Immunoblotting analysis is represented as floating bars with line representing the mean of n = 4 independent experiments ± SD, where the densitometric intensity was calculated with ImageJ software and expressed as arbitrary units (AU). §p < 0.001 *vs.* Ctr; **p < 0.01 *vs.* IL-6.

Given the key role of mitochondrial ROS in promoting both inflammatory responses and the induction of autophagy 30, we explored the effects of IL-6 and PCSK9i on ROS accrual and membrane potential in mitochondria. As evidenced by the staining with a fluorescent dye labelling mitochondrial ROS, IL-6 promoted a sharp increase in ROS levels, an effect prevented by PCSK9i pre-treatment (**Figure 3A-C**). Consistently, IL-6 also impaired the mitochondrial membrane potential of EC while PCSK9i hampered such increase, as indicated by labelling with a selective fluorescent dye (**Figure 3D-F**). In addition, these effects were accompanied by a modulation in the expression level of SIRT3, which was increased by IL-6 but returned to baseline with PCSK9i co-treatment (**Figure 3G**).

### SIRT3 silencing denies the protective effects of PCSK9i on inflammation, oxidative stress, and autophagy

**Figure 4:**
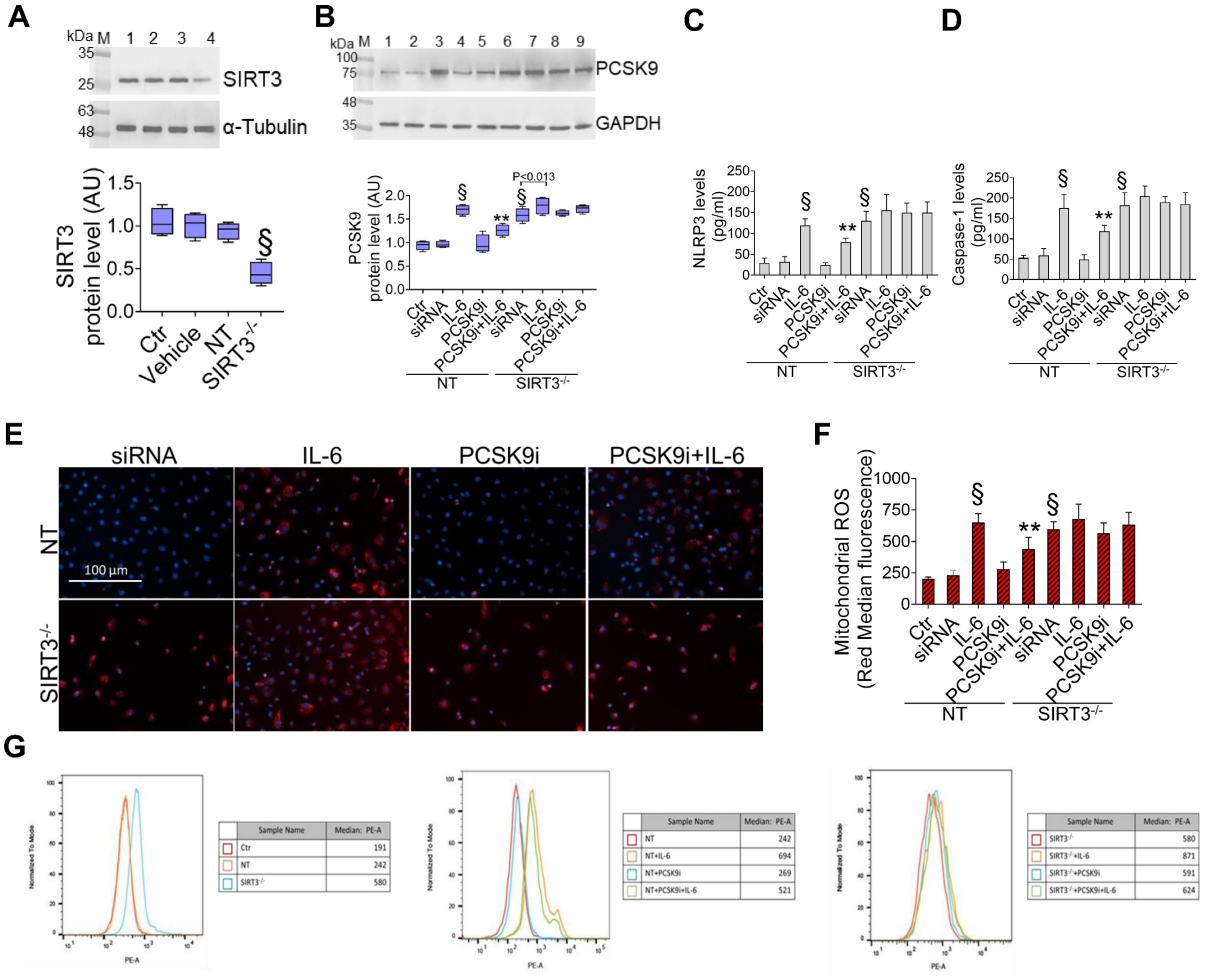
**Suppression of SIRT3 abolishes the effects of PCSK9i effects on inflammation and mitochondrial ROS. (A)** Representative cropped blots with relative immunoblotting analysis of SIRT3 protein levels in EC treated with the empty transfection reagent (Vehicle) or transfected with NT-siRNA (NT) or with SIRT3 siRNA (SIRT3^-/-^). Lane 1 = Ctr; lane 2 = Vehicle; lane 3 = NT; lane 4 = SIRT3^-/-^; M = weight markers (G266, Applied Biological Materials Inc.). **(B)** Representative cropped blots with relative immunoblotting analysis of PCSK9 protein levels, assessment of **(C)** NLRP3 and** (D)** caspase-1 content and **(E)** representative images by fluorescence microscopy and **(F,G)** FACS analysis of mitochondrial ROS evaluated in EC transfected with NT-siRNA (NT) or with SIRT3 siRNA (SIRT3^-/-^) and then exposed for 24h to 20 ng/mL IL-6 (NT or SIRT3^-/-^+IL-6), to 100 µg/mL PCSK9i (NT or SIRT3^-/-^+PCSK9i) or to 100 µg/mL PCSK9i for 8h before exposure to 20 ng/mL IL-6 for 24h (NT or SIRT3^-/-^+PCSK9i+IL-6). Control cells (Ctr) were maintained in complete culture medium with the corresponding highest volume of HBSS-10 mM Hepes. Scale bars = 100 µm. Lane 1 = Ctr; lane 2 = NT; lane 3 = NT+IL-6; lane 4 = NT+PCSK9i; lane 5 = NT+PCSK9i+IL-6; lane 6 = SIRT3^-/-^; lane 7 = SIRT3^-/-^+IL-6; lane 8 = SIRT3^-/-^+PCSK9i; lane 9 = SIRT3^-/-^+PCSK9i+IL-6; M = weight markers (G266, Applied Biological Materials Inc.). Immunoblotting analysis is represented as floating bars with line representing the mean of n = 4 independent experiments ± SD, where the densitometric intensity was calculated with ImageJ software and expressed as arbitrary units (AU). §p < 0.001 *vs.* NT; **p < 0.01 *vs.* NT+IL-6.

**Figure 5:**
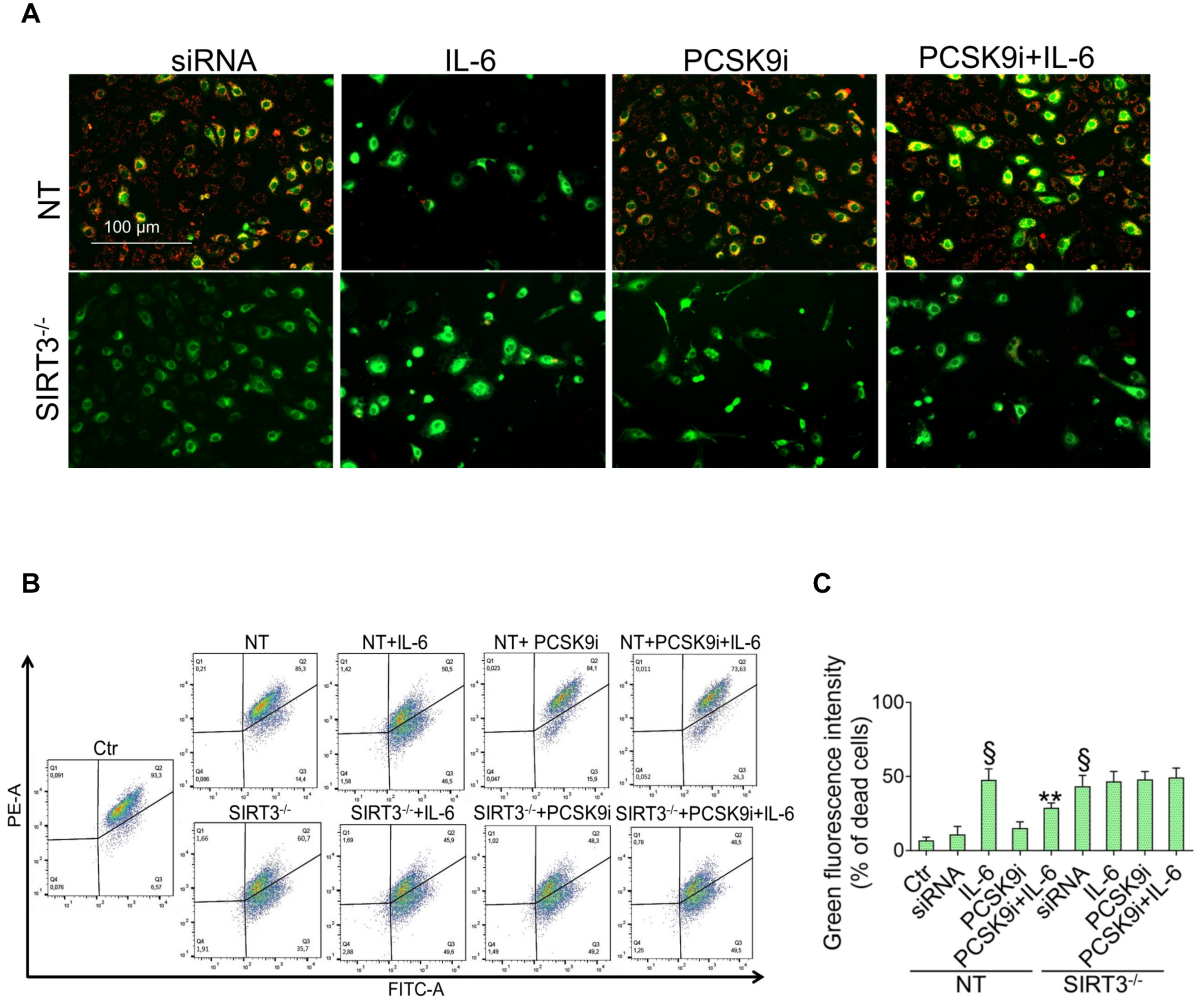
**Downregulation of SIRT3 decreases the ability of PCSK9i to impede mitochondrial depolarization. (A)** Representative images by fluorescence microscopy and **(B,C)** flow cytometry analysis of mitochondrial membrane potential detection, expressed as fluorescence intensity ± SD of n = 3 experiments, in EC transfected with NT-siRNA (NT) or with SIRT3 siRNA (SIRT3^-/-^) and then exposed for 24h to 20 ng/mL IL-6 (NT or SIRT3^-/-^+IL-6), to 100 µg/mL PCSK9i (NT or SIRT3^-/-^+PCSK9i) or to 100 µg/mL PCSK9i for 8h before exposure to 20 ng/mL IL-6 for 24h (NT or SIRT3^-/-^+PCSK9i+IL-6). Control cells (Ctr) were maintained in complete culture medium with the corresponding highest volume of HBSS-10 mM Hepes. Scale bars = 100 µm. §p<0.001 *vs.* NT; **p < 0.01 *vs.* NT+IL-6.

**Figure 6:**
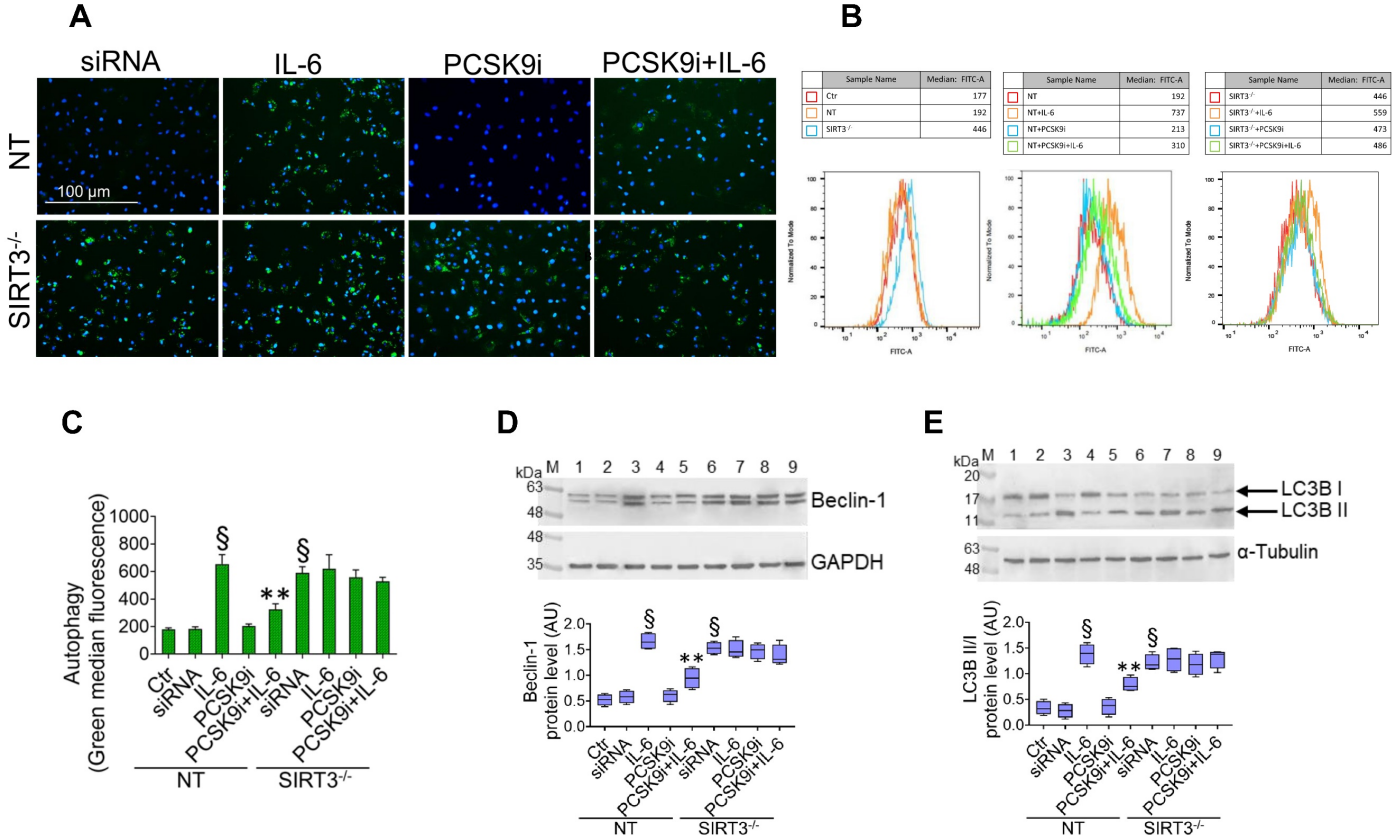
**SIRT3 suppression denies the ability of PCSK9i to counteract autophagy. (A)** Representative images by fluorescence microscopy and **(B,C)** flow cytometry analysis of green detection reagent, quantified as median fluorescence intensities, and representative cropped blots with relative immunoblotting analysis of **(D)** beclin-1 and **(E)** LC3B II/I protein levels in EC transfected with NT-siRNA (NT) or with SIRT3 siRNA (SIRT3^-/-^) and then exposed for 24h to 20 ng/mL IL-6 (NT or SIRT3^-/-^+IL-6), to 100 µg/mL PCSK9i (NT or SIRT3^-/-^+PCSK9i) or to 100 µg/mL PCSK9i for 8h before exposure to 20 ng/mL IL-6 for 24h (NT or SIRT3^-/-^+PCSK9i+IL-6). Control cells (Ctr) were maintained in complete culture medium with the corresponding highest volume of HBSS-10 mM Hepes. Scale bars = 100 µm. Lane 1 = Ctr; lane 2 = NT; lane 3 = NT+IL-6; lane 4 = NT+PCSK9i; lane 5 = NT+PCSK9i+IL-6; lane 6 = SIRT3^-/-^; lane 7 = SIRT3^-/-^+IL-6; lane 8 = SIRT3^-/-^+PCSK9i; lane 9 = SIRT3^-/-^+PCSK9i+IL-6; M = weight markers (G266, Applied Biological Materials Inc.). Immunoblotting analyses are represented as floating bars with line representing the median of n = 4 independent experiments ± SD, where the densitometric intensity was calculated with ImageJ software and expressed as arbitrary units (AU). §p < 0.001 *vs.* NT; **p < 0.01 *vs.* NT+IL-6.

Given the results relative to SIRT3 modulation and considering the known role played by this protein in the regulation of inflammatory responses, autophagy, and oxidative stress 27,28, we explored whether SIRT3 silencing (**Figure 4A**) reverted the beneficial effects of PCSK9i on these phenotypes. Forced downregulation of SIRT3 *per se* phenocopied the effect of IL-6 and blunted the protective effect provided by PCSK9i against the IL-6 induced expression of PCSK9 (**Figure 4B**), the soluble levels of NLPR3 (**Figure 4C**), and of caspase-1 (**Figure 4D**), as well as on the induction of mitochondrial ROS (**Figure 4E-G**), and of mitochondrial membrane potential (**Figure 5A-C**). Similarly, neither treatment with IL-6 nor PCSK9i have significant effects on the modulation of autophagy when SIRT3 was silenced in EC (**Figure 6A-C**), an observation corroborated by the expression of Beclin-1 (**Figure 6D**) and of the ratio LC3BII/I in this setting (**Figure 6E**).

### Correlations among PCSK9, SIRT3, IL-1β, LC3B II/I, and IL-6

To gain preliminary insights about the relevance of these findings in patients, we assessed the expression of IL-6, PCSK9, SIRT3, IL-1β, and of the ratio LC3B II/I within the plaques of patients undergoing carotid endarterectomy and not treated with PCSK9i (n=277, characteristics in **Supplementary Table 1**) to explore a possible correlation between these variables through linear regression analyses. As shown in **Table 1**, the expression of PCSK9 was inversely related to that of SIRT3 while positively correlating with IL-6, IL-1β, and the ratio LC3B II/I. Consistently, IL-6 showed a positive correlation with LC3B II/I and a borderline relationship with IL-1β, overall supporting a framework where PCSK9 and SIRT3 are correlated each other and associated with low-grade inflammation and autophagy also in human samples.

## Discussion

PCSK9i represent an additive or an alternative option to attain LDL cholesterol targets when patients are statin-intolerant or not-at-target with maximal dosage with other lipid-lowering drugs 31. Beyond lowering LDL cholesterol, preclinical data evidenced a plethora of beneficial effects with PCSK9 inhibition, especially in EC. Of note, these effects were suggested to be independent of LDL cholesterol levels and mediated by a direct role of PCSK9 protein 3-6, 32. Here we substantiate these observations by showing that PCSK9i exert anti-inflammatory, anti-autophagic, and antioxidant effects in EC exposed to IL-6, a potent cytokine linked to the development of CVD. In our model, these beneficial effects were mediated, at least in part, by SIRT3, which downregulation largely blunted the protection conferred by PCSK9i. Consistently, we found a negative correlation between the expression of PCSK9 protein and SIRT3 within the plaques of patients, while IL-6, IL-1β, and the ratio LC3B II/I correlated positively with PCSK9 and negatively with SIRT3, overall supporting a framework where PCSK9i might eventually attenuate a pathological cascade instigated by low-grade inflammation. To our knowledge, this is the first study hypothesizing such framework and in particular showing a role for SIRT3 in the cardioprotective effects promoted by PCSK9i.

Previous studies provided mixed results relatively to an anti-inflammatory effect of PCSK9i in humans. Indeed, hs-CRP, a known marker of low-grade inflammation, is not affected by treatment with PCSK9i 33. On the other hand, selected reports showed that treatment with PCSK9i might decrease circulating levels of IL-6 and of other cytokines 34, 35. Interestingly, also IL-6 inhibition has been reported to decrease circulating levels of PCSK9 protein 15, possibly suggesting a bidirectional relationship. Alternatively, it has been hypothesized that PCSK9i ameliorate low-grade inflammation at the local level within the vasculature without affecting systemic inflammation 36. Whatever the case, data presented here support a potent anti-inflammatory effect for PCSK9i. Mechanistically, it was suggested that PCSK9 protein promotes mitochondrial DNA damage to activate NLRP3 inflammasome signalling 37. Similarly, SIRT3 deficiency is known to impair mitochondrial respiration and promote inflammasome activation 38. These evidences are in line with our data showing the inability of PCSK9i to blunt inflammasome activation when SIRT3 is suppressed.

The roles of PCSK9 protein in the induction of autophagy and of PCSK9i in the attenuation of the same phenotype were evidenced in cardiomyocytes and in microvascular cells 5,6. Of note, these findings were substantiated in heart specimens from patients with a recent myocardial infarction 5. At the molecular level, it was suggested that PCSK9 might promote autophagy from at least two pathways, both of which are instigated by the accumulation of ROS within mitochondria 5,6. Given the known role of SIRT3 in suppressing oxidative stress in this organelle 38-40 and, more broadly, autophagy 26,41, it is conceivable that SIRT3 downregulation denied the effect of PCSK9i on this phenotype.

Recombinant human PCSK9 protein was previously shown to induce a dose-dependent accumulation of ROS in multiple cell types, including EC 42. Of note, this possible relationship was also observed in humans, since patients with higher PCSK9 levels have also an increased burden of plasmatic ROS 43. Consistently, treatment with PCSK9i has been demonstrated to counteract the damage caused by H2O2 in EC 44, while patients treated with PCSK9i for 6 months also showed a marked decrease in the levels of oxidative stress 45. Mechanistically, it was suggested that PCSK9 protein promote the expression of the NADPH oxidases subunits p47phox and gp91phox to foster oxidative damage 32. Of note, SIRT3 was previously suggested to regulate these same subunits in a model of Angiotensin-II-induced ROS formation 46, an observation compatible with our findings.

Our study has some limitations. Indeed, we did not explore whether the modulation of autophagy is beneficial or detrimental for EC function in our model. Broadly, both hypotheses have been advanced, according to the different models used 51, 52. Thus, more research is needed to establish if the anti-autophagic effects of PCSK9i are relevant for their cardioprotective properties. More importantly, we have not tested if plaque specimens from patients treated with a PCSK9i have a lower burden of inflammation, autophagy, and oxidative stress compared with those from patients treated with other lipid-lowering drugs, an aspect that must be assessed in future studies to substantiate the clinical relevance of the findings reported here.

## Conclusions

In summary, here we showed that PCSK9i are able to counteract the deleterious effects of IL-6 on the activation of inflammatory pathways, the instigation of autophagy, and the induction of oxidative stress in EC and that SIRT3 suppression abolishes the protective effects of PCSK9i. Consistently, we also demonstrated a correlation among PCSK9, SIRT3, IL-6, IL-1β, and the ratio LC3B II/I in plaques of patients undergoing carotid endarterectomy. Albeit not proving causality, these findings are compatible with a framework where PCSK9i blunt a range of detrimental phenotypes instigated by low-grade inflammation in the endothelium (summarized in the **Graphical Abstract**). These data further sustain the argument that PCSK9i might provide CV benefit also beyond LDL-cholesterol lowering, providing an additional rationale for its use in patients with high cardiovascular risk independently of their LDL-cholesterol levels 2,47,48. In addition, these observations encourage further research about the role of SIRT3 as a potential mediator of the effect of PCSK9i and, more broadly, as a candidate cardioprotective molecule.

## Supplementary Material

Supplementary figures and table.

## Figures and Tables

**Table 1:** ** Relationship among IL-6, SIRT3, IL-1β, the ratio LC3B II/I, and PCSK9 in plaque specimens.** Results of the linear regression analyses showing a correlation among the expression of the different proteins assessed in plaque specimens obtained from patients undergoing carotid endarterectomy (n=277) and not treated with PCSK9i. Beta coefficient, r2, the relative p values, and 95% confidence interval (CI) are shown

		PCSK9	SIRT3	IL-6	IL-1β	LC3BII/I
PCSK9	B	-	-0,751	0,236	0,090	0,125
	R^2^	-	-0,476	0,280	0,133	0,130
	p-value	-	<0,001	<0,001	0,002	0,002
	95% CI	-	-0,900; -0,603	0,164;0,308	0,034;0,146	0,045;0,205
SIRT3	B		-	-0,014	-0,077	-0,096
	R^2^		-	-0,026	-0,180	-0,157
	p-value		-	0,612	<0,001	<0,001
	95% CI		-	-0,067;0,040	0,0115;0,039	-0,151; -0,041
IL-6	B			-	0,077	0,168
	R^2^			-	0,096	0,147
	p-value			-	0,085	0,008
	95% CI			-	-0,011;0,164	0,044;0,292
IL-1β	B				-	-0,302
	R^2^				-	-0,212
	p-value				-	<0,001
	95% CI				-	-0,468; -0,136
LC3BII/I	B					-
	R^2^					-
	p-value					-
	95% CI					-

## Abbreviations

PCSK9: proprotein convertase subtilisin-kexin type 9
SIRT3: NAD-dependent deacetylase sirtuin-3
NLRP3: NLR family pyrin domain containing 3
IL-1β: Interleukin 1 β
TNFα: Tumour necrosis factor α
NF-kB: Nuclear factor kappa-light-chain-enhancer of activated B cells
IFN-γ: Interferon gamma
ROS: Reactive Oxygen Species
p62: Sequestosome-1
ATG5: Autophagy related 5
LC3B II/I: Microtubule-associated proteins 1A/1B light chain 3B
