# Supplementary material for: SIRT3 mediates the effects of PCSK9 inhibitors on inflammation, autophagy, and oxidative stress in endothelial cells
Source: Theranostics. 2023 Jan 1;13(2):531–42. doi: 10.7150/thno.80289 (PMC9830434; doi:10.7150/thno.80289)
Supplement: Supplementary file 1 — Supplementary figures and table. [file thnov13p0531s1.pdf]

Supplementary Material For

SIRT3 mediates the effects of PCSK9 inhibitors on inflammation, autophagy, and oxidative stress in endothelial cells

Nunzia D'Onofrio[1+], Francesco Prattichizzo[2+], Raffaele Marfella[3,4], Celestino Sardu[3], Elisa Martino[1], Lucia Scisciola[3], Lorenza Marfella[3], Rosalba La Grotta[2], Chiara Frigé[2], Giuseppe Paolisso[3,4], Antonio Ceriello[2*], Maria Luisa Balestrieri[1*].

[1]Department of Precision Medicine, the University of Campania "Luigi Vanvitelli" Italy;
[2] IRCCS MultiMedica, Via Fantoli 16/15, 20138, Milan, Italy;
[3]Università degli Studi della Campania "Luigi Vanvitelli", Piazza Luigi Miraglia 2, 80138, Naples, Italy;
[4]Mediterranea Cardiocentro, 80122, Naples, Italy.

+ These authors contributed equally to the manuscript

***Address correspondence to:**

Maria Luisa Balestrieri
Università degli Studi della Campania "Luigi Vanvitelli"
Via L. De Crecchio 7, 80138
Naples, Italy
marialuisa.balestrieri@unicampania.it
Antonio Ceriello
IRCCS MultiMedica, PST
Via Fantoli 16/15, 20138
Milan, Italy
antonio.ceriello@hotmail.it

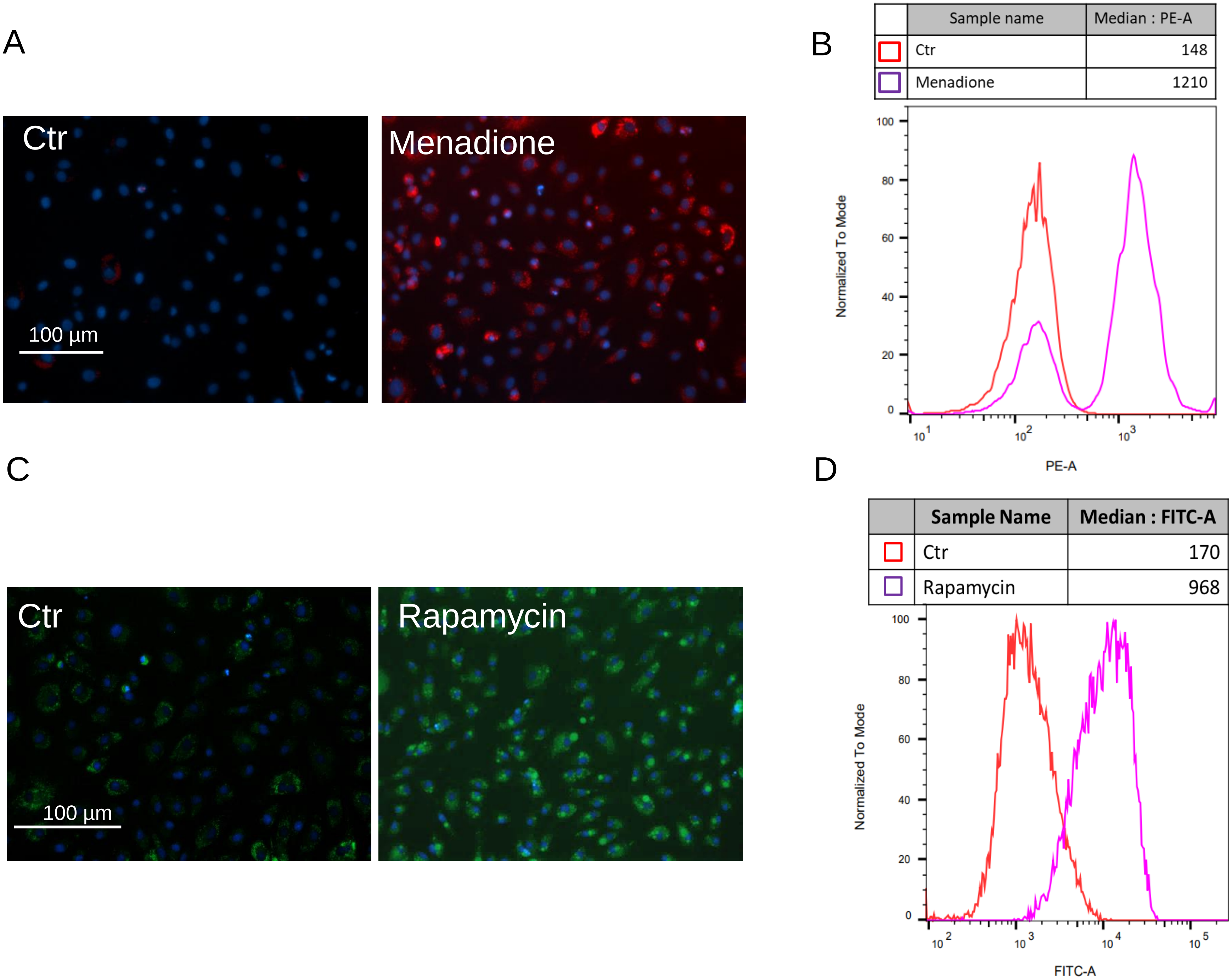

Supplementary Figure 1. Positive controls for experiments assessing induction of mitochondrial ROS and autophagy. (A) Representative images and (B) cytometer analysis, expressed as red fluorescence median, of ROS detection in EC treated for 30 min with 50 µM menadione, the ROS inducer, or with the corresponding highest volume of HBSS-10 mM Hepes (Ctr) and stained with MitoSOX Red probe. (C) Representative images and (D) cytometer analysis, expressed as green fluorescence median, of autophagy detection in EC treated for 16h with 1 µM rapamycin, the autophagy inducer, or with the corresponding highest volume of HBSS-10 mM Hepes (Ctr) and stained with Green detection reagent. Scale bars = 100 µm.

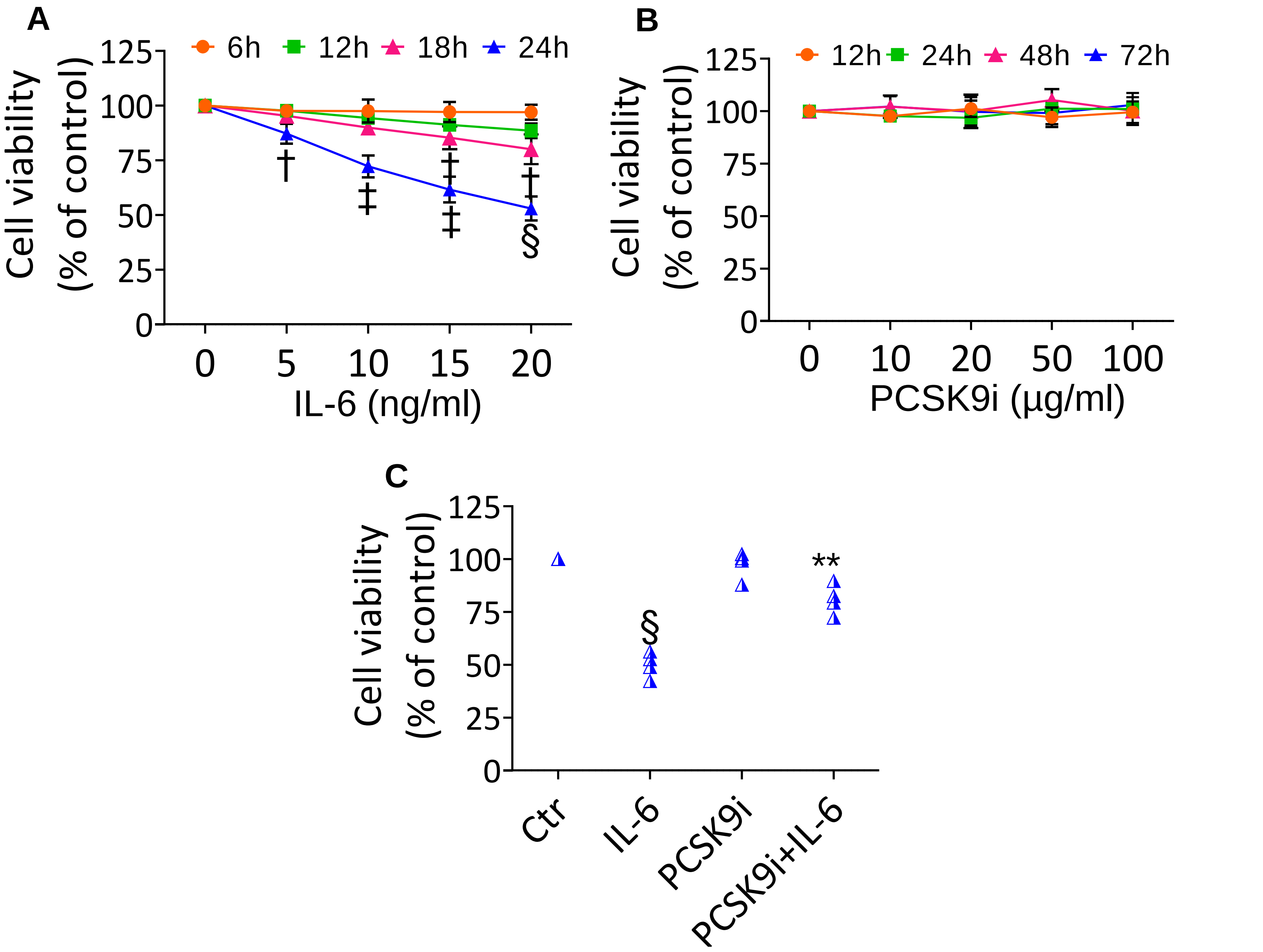

Supplementary Figure 2. IL-6 and PCSK9i dose-response experiments. EC viability after exposure to different concentrations of (A) IL-6 (0-20 ng/mL) up to 24, to PCSK9i (0-100 µg/mL) up to 72h and (C) treated for 24h with 20 ng/mL IL-6 (IL-6), with 100 µg/mL PCSK9i (PCSK9i) or pre-treated for 8h with 100 µg/mL PCSK9i before being exposed to 20 ng/mL IL-6 for 24h (PCSK9i+IL-6). Control cells (Ctr or 0 mM) were treated with the corresponding highest volume of Hanks' balanced salt solution (HBSS)-10 mM Hepes. Cell viability was assessed by Cell Counting Kit-8 assay and reported as % of control. †$p<0.05$ vs 0 ng/ml; ‡$p<0.01$ vs 0 ng/ml; §$p<0.001$ vs 0 ng/ml or Ctr; **$p<0.01$ vs IL-6.

Supplementary Table 1. Characteristics of study patients.

Variable	
N	277
Sex (male), n (%)	136 (49.1)
Age, years	65.3±5.9
BMI, kg/m^2	28±1.8
Glucose, mg/dl	162.1±53.4
HbA1c, %	7.6±4.1
Cholesterol, mg/dl	160±17.2
LDL-cholesterol, mg/dl	98.4±24.1
HDL-cholesterol, mg/dl	40.2±3.8
Triglycerides, mg/dl	177±23.8
Hypertension, n (%)	177 (63.9)
Previous cardiovascular event, n (%)	129 (46.6)
Diabetes, n (%)	56 (20.2)
Smoking, n (%)	52 (18.8)
Statins, n (%)	248 (89.5)
Ezetimibe, n (%)	74 (26.7)

Data are presented as mean ± SD or as number (%).BMI = body mass index; HbA1c= Haemoglobin A1c; HDL= high-density lipoprotein; LDL = low-density lipoprotein.
